# The Scanning TMR Microscope for Biosensor Applications

**DOI:** 10.3390/bios5020172

**Published:** 2015-04-02

**Authors:** Kunal N. Vyas, David M. Love, Adrian Ionescu, Justin Llandro, Pratap Kollu, Thanos Mitrelias, Stuart Holmes, Crispin H. W. Barnes

**Affiliations:** 1 Cavendish Laboratory, Department of Physics, University of Cambridge, JJ Thomson Avenue, CB3-0HE Cambridge, UK; E-Mails: ai222@cam.ac.uk (A.I.); jl286@cam.ac.uk (J.L.); pk419@cam.ac.uk (P.K.); tm10007@cam.ac.uk (T.M.); s.holmes@crl.toshiba.co.uk (S.H.); chwb101@cam.ac.uk (C.H.W.B.); 2 Cambridge Biomagnetics Ltd., St John’s Innovation Centre, Cowley Road, CB4-0WS Cambridge, UK; 3 Toshiba Research Europe Ltd., Cambridge Research Laboratory, 208 Cambridge Science Park, Milton Road, CB4-OGZ Cambridge, UK

**Keywords:** magnetic carriers, TMR-scanning microscope, 3D magnetic stray field mapping, domain wall characterisation

## Abstract

We present a novel tunnel magnetoresistance (TMR) scanning microscope set-up capable of quantitatively imaging the magnetic stray field patterns of micron-sized elements in 3D. By incorporating an Anderson loop measurement circuit for impedance matching, we are able to detect magnetoresistance changes of as little as 0.006%/Oe. By 3D rastering a mounted TMR sensor over our magnetic barcodes, we are able to characterise the complex domain structures by displaying the real component, the amplitude and the phase of the sensor’s impedance. The modular design, incorporating a TMR sensor with an optical microscope, renders this set-up a versatile platform for studying and imaging immobilised magnetic carriers and barcodes currently employed in biosensor platforms, magnetotactic bacteria and other complex magnetic domain structures of micron-sized entities. The quantitative nature of the instrument and its ability to produce vector maps of magnetic stray fields has the potential to provide significant advantages over other commonly used scanning magnetometry techniques.

## 1. Introduction

In modern biosensors, particularly in microfluidic-based platforms, the incorporation of micron- and nano-scale carriers has a wide range of applications and benefits to address specific platform requirements [1]. For suspension assay technologies [2], the identification of functionalised carriers can, for instance, be carried out through the use of optical barcodes [3,4,5], fluorescence wavelengths [6] and specified binding sites [7,8], amongst others. An unconventional approach to microcarrier identification in bioassays is the use of magnetic barcodes, which also provides significant scaling advantages [9]. Moreover, magnetic carriers in general are becoming increasingly relevant to areas, such as cancer treatment and drug discovery, where functionalised magnetic nanoparticles are used for hyperthermia treatment of cancers [10] and for the characterisation of protein-antigen interactions on sensor surfaces [11], respectively. It is clear that magnetic carriers continue to play key roles in many modern clinical treatments and biosensing platforms [12,13,14].

**Figure 1:**
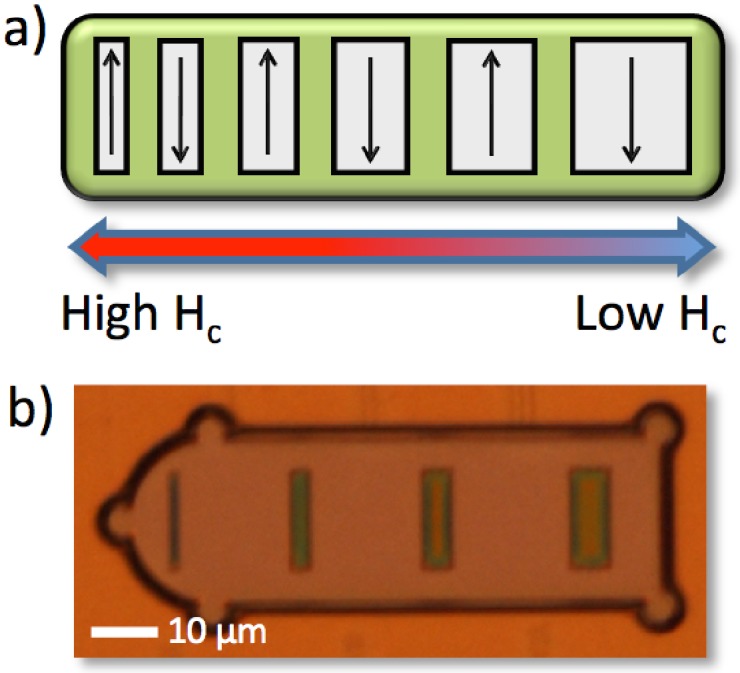
(**a**) Illustration of coercivity (*H_c_*) tuned bit encoding, where the varied aspect ratios of each bit define unique field values at which the magnetisation reversal occurs; (**b**) Microscope image of a four-bit microcarrier.

Previously, we have demonstrated the use of coercivity tuning of magnetic elements for the encoding of suspended microcarriers (or “tags”), providing unique binary codes, by using sequences of applied magnetic field pulses [15]. Figure 1a,b illustrate how the variation of the aspect ratio for each magnetic “bit” determines its coercivity value (*H_c_*) at which the magnetisation reversal (transition between binary states 1 and 0) occurs. This allows for the mass fabrication of nominally-identical tags, where each bit’s state is individually addressable using oscillating external magnetic fields when encoding from the highest to the lowest coercivity value. It has also been demonstrated that these microcarriers can be functionalised on two distinct surfaces (*i.e.*, at the thiol and epoxy groups) with target molecules for bioassay applications [16] and subsequently identified, once positive binding is detected, using in-flow magnetic read-out via an incorporated tunnel magnetoresistance (TMR) sensor [17,18]. We have shown previously [17] that the microcarriers can be detected in a microfluidic channel at flow speeds of 3.6 mm/s at a height of 5 *µ*m above the sensor.

For the successful development of this platform, the detailed characterisation and understanding of the magnetic behaviour of our barcode design has been crucial. In order to do so, we have constructed a TMR scanning microscope that is capable of mapping out the stray fields of immobilised magnetic elements in 3D and in various modes. Here, we present in detail the instrument design, characterisation and its overall capabilities, by demonstrating how, for instance, raster scans can map out the stray field of immobilised magnetic elements in high resolution and in multiple dimensions. In comparison to traditional magnetometry techniques, such as magnetic force microscopy (MFM) or magneto-optical Kerr effect (MOKE) measurements, the TMR microscope is able to provide quantitative magnetic field amplitudes with 3D spatial resolution. The highly modular and adaptable nature of this set-up makes it particularly relevant for the study of various different magnetic carrier types used in modern biosensing technologies.

## 2. Instrument Design

A key feature of the TMR microscope is the use of an Anderson loop [19], which unlike a standard Wheatstone bridge is more suitable for low noise AC measurements of a lock-in technique and provides a linear response to changes in the impedance. In the Anderson loop measurement system, the potential difference across the impedance is not amplified through the use of passive components as in the Wheatstone bridge, but through the use of powered amplifiers. Only resistors and capacitors were used in our initial bridge design, and the voltage applied to the Wheatstone bridge is the source of both the current through the measured impedance and the “amplified” signal that we measured.

**Figure 2:**
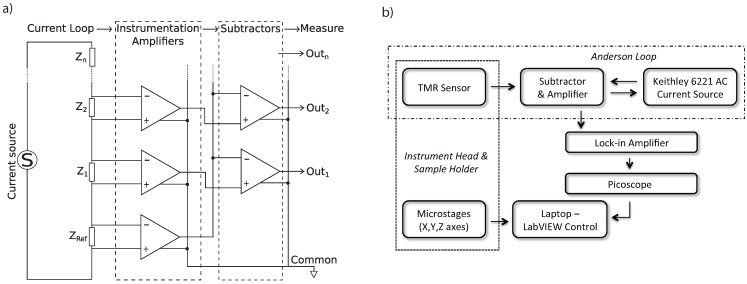
(**a**) The Anderson loop measurement circuit, developed by NASA [19], is a precision current source used to supply a series of impedances, *Z*_1_ to *Z_n_*, and a reference impedance, *Z_ref_* . The potential difference across each impedance is amplified by a set of instrumentation amplifiers. The reference signal is subtracted using a set of differencing amplifiers to give us a series of outputs, proportional to the difference between the reference impedance and each measured impedance, respectively; (**b**) Block diagram showing how all of the various pieces of equipment are connected. Note that the lock-in frequency can be defined by the Keithley power source using a trigger line or by its own internal oscillator.

Figure 2a shows how a generalised Anderson loop measurement system works. In our pre-amplifier design, a Keithley 6221 AC precision current source is used to supply a small current (typically 1.5 *µ*A) through our Anderson loop, which contains a reference impedance and up to four TMR sensors. The potential difference across the reference impedance and any two of the sensors connected is amplified by a set of instrumentation amplifiers, which amplify the potential difference between two inputs, as illustrated in Figure 2a. After the amplification stage, the reference signal is subtracted from the sensor signal using an op-amp (INA105OKP) before being passed on to our lock-in amplifier. The reference is subtracted in order to negate any fluctuations in the current passing through our loop and to provide as small a background level as possible for any signals that we detect. The impedances of the purchased TMR sensors, from Micro Magnetics Inc. (http://www.micromagnetics.com), vary hugely, typically from 5 to 35 kΩ; therefore, we have incorporated the ability to switch between five different reference impedances: *Z_ref_* = 0.1/2.1/5.1/10.1/20.1 kΩ. Whilst the reference signal has a fixed amplification of 10×, the sensor amplification can be adjusted to match the reference signal amplitude through the tuning of a variable resistor, so that two sensors with differing impedances can be matched with the same reference impedance. In our pre-amp, the variable resistor can be tuned from 1 to 6 kΩ, corresponding to a gain range of 21–4.3, respectively. This effectively means that we can match an impedance that is between half as big and twice as big as each reference impedance.

The Anderson loop electronics are connected to a custom-made sensor chip holder, using a sprung 11-pin gold connector. Six grounded pins are interleaved with the five pins used to carry signals from our sensor. Since TMR sensors are particularly sensitive to electrostatic discharge, adjacent lines will be shorted in the case of high voltages through antiparallel BAT54J high-speed diodes. In addition to the ability to measure the impedance between any two sets of lines, we also have the option to short-circuit them. This means that we can bypass blown up sensors or broken wire bonds. Finally, the two outputs, Channel 1 and Channel 2, of our pre-amp have a low-pass filter that blocks high-frequency noise.

After the subtracter, the amplified signal is sent to an EG&G Instruments 7265 DSP lock-in amplifier, which outputs a DC signal proportional to the signal received at the operating frequency. This signal is measured by a PicoScope 3204 digital oscilloscope, so that it can be processed by a laptop. A block diagram of our instrument can be seen in Figure 2b. All of the equipment in the measurement set-up is powered by an APC ES400 uninterruptible power supply, which was found to significantly reduce the noise entering the system from the main’s ground and power.

A representative sensitivity characterisation of the used TMR sensor batch is presented in a previous publication [20], with an estimated sensitivity of *S_se_* ≈ 175 *µ*V/Oe with a minimum detectable field of 0.2–1.3 Oe, varying from sensor to sensor. This compares well with a spin valve-based scanning magnetoresistance microscope [21], which specifies a sensitivity of 14.8 *µ*V/Oe and shows measured magnetic fields down to ∼1.3 Oe.

**Figure 3:**
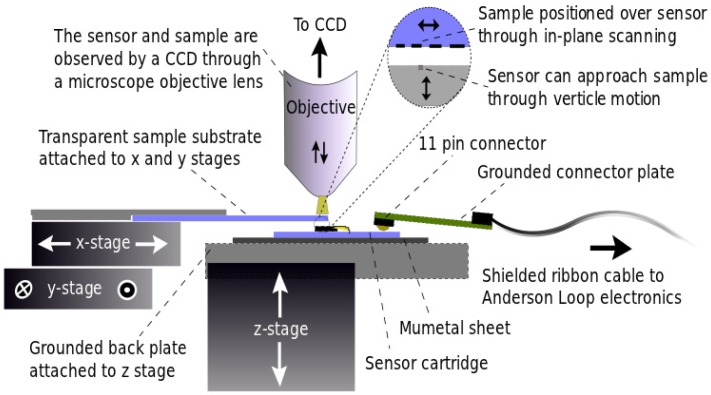
The figure illustrates how the instrument mechanics operate. The sample and sensor can be translated in all directions with respect to each other using three micro-stages that operate in orthogonal directions; *x*, *y* and *z*. The micro-stages, to which the sensor and sample are attached, are fixed to the microscope stage in order to enable the focussing of the objective. This allows either the sample or the sensor to be brought into the focal plain. When the vertical separation between them is small enough, as it is during measurements, both can be viewed at the same time.

The mechanics and optics of the instrument consist of a Leica DMLM microscope, three T25-D/M micro-stages from Elliot Scientific (now supported by Thorlabs), three TDC001 T-Cube DC servo motor drivers (Thorlabs), a CCD sensor and a custom-built sensor head holder and are best understood graphically by viewing Figure 3. The sample is placed/fabricated on a transparent substrate and is mounted upside down. It is attached to the *x*- and *y*-stages and can therefore be moved around in-plane. The sensor head is attached to a *z*-stage, which allows the sensor to approach the sample from below. This whole contraption sits inside a microscope, allowing us to view the sample and the sensor through the transparent sample substrate. The micro-stages control the relative *x*, *y* and *z* positions of the sensor and sample, whilst the microscope stage can be used to bring different planes into focus. The CCD-sensor is attached to the microscope to negate the need to constantly look through the eyepieces. The micro-stages are controlled by the laptop via their individual drivers (T-Cube) supplied by Thorlabs.

A program and corresponding user interface were developed using National Instruments LabVIEW 8.6 to control the entire instrument and to collect data with various levels of automation. In practice, this involved controlling and reading the output of the PicoScope, the three micro-stages and the CCD simultaneously to form an integrated and fully functioning program.

## 3. Characterisation

### 3.1. Frequency Response of the Pre-Amplifier

**Figure 4:**
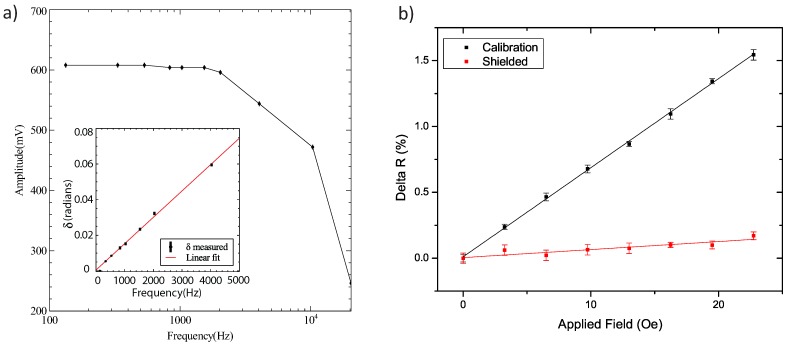
(**a**) The peak-peak voltage signal observed when measuring the reference through our pre-amplifier. The reference impedance was 20.1 kΩ and the current was ± 1.5 *µ*A. The inset shows the phase difference between the reference and sensor signals as a function of frequency; (**b**) The percentage change in resistance, ∆*R* (%), due to an in-plane field is shown in black. The data in red show the same measurement after a sheet of mu-metal is added in between the glass cartridge and the scanner head.

The frequency response of the pre-amplifier was investigated using the measurement channel with the sensor bypassed. Thus, the output signal, measured after the subtracter, only depends on the reference impedance, which was set to 20.1 kΩ. An AC current with an amplitude of ± 1.5 *µ*A was supplied, and the peak-peak voltage amplitude at the channel output was recorded as a function of frequency using an oscilloscope. The measurement is shown in Figure 4a.

The initial value of our peak-peak signal, remembering that the voltage is amplified with a gain of 10, closely fits the expected value of 603 mV. The signal drops off dramatically at high frequencies due to the low-pass filters incorporated into our electronics. The pre-amp is designed to work at frequencies less than 2 kHz, and noise above this frequency is filtered out. The measurement confirms this and gives us the frequency response of our pre-amp.

The above characterisation is repeated, but this time without bypassing the TMR sensor. For each frequency, we carefully adjust the gain applied to the sensor signal in order to cancel and reduce the reference as much as possible. The peak-peak amplitude of the residual signal is then measured as a function of frequency. To explain what we are measuring, consider the waveform, *ψ*, obtained by the cancellation of two sinusoidal signals of equal amplitude, *A*, with a phase difference of *δ*:

*ψ* = *A*(sin *x* − sin (*x* + *δ*)) = *A*(sin *x* − (sin *x* cos *δ* + sin *δ* cos *x*)). (1)

Therefore, when the phase difference is small:

*ψ* = −*Aδcosx*. (2)

By finding the peak-peak value of the minimum residual signal, we are measuring *A* · *δ*, where the amplitude, *A*, has already been measured in Figure 4a. Hence, the phase difference *δ* between our reference and sensor can be found and is plotted as a function of frequency in the Figure 4a inset.

It can be seen that the phase difference increases linearly with frequency at a rate of (1.48 ± 0.01) × 10^−5^ radians/Hz. This linear trend corresponds to an effective time lag, ∆*t*, between the two signals of:

(3) $$\text{Δ}t=\frac{1}{2\pi }\frac{d\delta }{df}=(2.35\pm 0.02)\mu s.$$

This effective lag is due to slight differences in the inductances and capacitances in the paths that the two signals must take. The sensor signals must pass through wire bonds, the base chip and a ribbon cable, whilst the reference resides inside our pre-amp box. The printed circuit board (PCB) design itself can cause the signals to experience different complex impedances. Since we are using low frequencies and are interested in signals over the period of many milliseconds rather than microseconds, the phase lag measured will not significantly impact our measurement. However, this analysis indicates that for a similar instrument operating at high frequencies (e.g., MHz range), one would need to redesign the PCB or possibly introduce a way to balance the complex impedances. The benefit of running at higher frequencies would be a reduction in the time constant of our measurement from the order of 100 ms to 100 *µ*s, allowing for high-speed operation. Another benefit of high-frequency operation is overcoming 1/*f* noise. Assuming then that we are Johnson noise limited, the theoretical minimum detectable field is determined by the following equation [21]:

(4) $$SNR=\frac{S_{se}\times H}{\sqrt{4k_{B}TR_{S}\Delta f}},$$

where *SNR* is the signal-to-noise ratio, *S_se_* is sensor sensitivity, *H* is the applied magnetic field, *k_B_* is the Boltzmann constant, *T* is temperature, *R_S_* is sensor resistance and ∆*f* is the bandwidth. Assuming a bandwidth of 1 Hz and a 10 *µ*A excitation current, the expected magnetic field sensitivity (*SNR* = 1) is 175 *µ*Oe. This is slightly higher than the theoretical limit published in [21] for an out-of-plane MR scanning system. In [21], the minimum detected field is 1.3 Oe, whilst our system detected fields as low as 0.25 Oe. Furthermore, as the current system operates at low frequencies, where we are 1/*f* -limited [22,23], we believe that there is significant scope for further improvement in the minimum detectable field.

### 3.2. Sensor Calibration and Mu-Metal Shield

A working TMR sensor is calibrated on our set-up with an in-plane magnetic field applied using an air cored Helmholtz coil. The voltage output of the lock-in amplifier is measured using the PicoScope. However, ideally, we do not wish to calibrate it by using this value, as it depends on the measurement parameters and lock-in settings. We therefore calibrate the sensor by using the percentage change in resistance of our sensor with respect to the field. This can be obtained using:

(5) $$\Delta R\left[\%\right]=100\times \frac{\Delta R\left[\Omega \right]}{R_{*}},$$

where *R_∗_* is the resistance of the sensor, which can be taken as the resistance of the reference, and:

(6) $$\Delta R\left(\Omega \right)=\frac{V_{measured}\cdot S_{li}}{10\cdot G\cdot I},$$

where *S_li_* is the sensitivity setting of the lock-in, *G* is the gain of our pre-amp, which can be taken as ∼10, *I* is the amplitude of the applied current and *V_measured_* is the voltage level measured on the PicoScope.

Figure 4b gives us the resulting calibration using the analysis above and a linear fit to the data yields the slope. The straight line fit gives us the calibration slope of 0.0676 ± 0.0008 %/Oe. After measuring the sensor’s response to the field, we add a mu-metal sheet below the glass cartridge. Whilst this does not fully enclose the sensor, adding a mu-metal plate in the plane in which we are measuring this close to the sensor (≈600 *µ*m) will attenuate external fields. The new slope is measured as 0.0061 ± 0.0011 %/Oe, showing an 11× attenuation of the field at the sensor position.

## 4. Detection of Magnetic Barcodes

### 4.1. One-Dimensional Stray Field Scans

**Figure 5:**
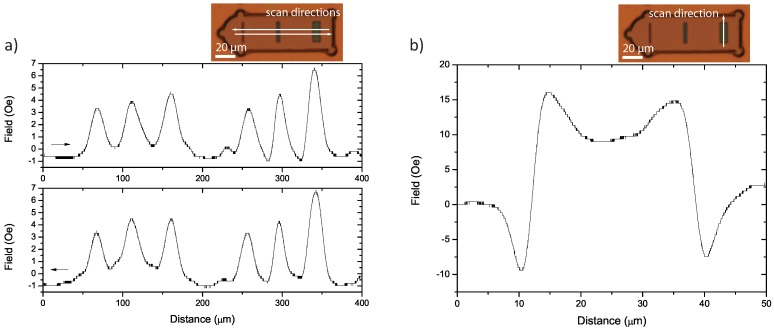
(**a**) Two three-bit tags are scanned in forward (above) and reverse (below) direction, showing a high level of reproducibility. A 10 *µ*m/s scan speed was used with a measurement time-constant of 500 ms. The vertical distance between the sample and sensor is ≈5 *µ*m; (**b**) A single magnetic element scanned along its long axis at 10 *µ*m/s with a 100 ms time constant. The scan shows that the signal level increases as you travel form the centre of the element to the edge, after which the signal changes sign before dying away. The sensitive axis the TMR sensor for (**a**) and (**b**) points along the length of the magnetic elements.

TMR line scans were taken of Co magnetic elements of varying aspect ratios (1:8, 1:5, 1:2.5), ∼20 nm thick, 20 *µ*m long and approximately 40 *µ*m apart. The samples were grown on a glass substrate, so that they could be simultaneously viewed using the optical microscope. Scans were taken, scanning across several magnetic elements, with the long, magnetically easy, axis of the elements perpendicular to the scan path and parallel to the sensitive direction of the TMR sensor. Figure 5a shows the field as measured by a TMR sensor as it was scanned at 10 *µ*m/s across two three-bit tags in the forward and reverse directions. A frequency of 833 Hz was used with a time constant of 500 ms.

The approximate spatial resolution of a scan is given by the larger of two values: the distance between sample and sensor, ∆*h*, or *v* · *τ* + *d*, where *v* is the scan speed, *τ* is the time-constant of the measurement and *d* is the diameter of the sensor. The larger of the two values should generally be used, and the resolution in this case is 7 *µ*m. The amplitude of the noise observed in the scan-background varies between 0.25 and 0.5 Oe, giving us for the highest peak an *SNR* of 15:1.

Along with the encouraging improvement in the *SNR* of the measurements, the control over the position of the elements, unlike during in-flow detection [17], allows the reproducibility of the measurement to be tested. This was done by reversing the scan direction and re-measuring along the same line, resulting in an almost identical measurement. For a higher resolution, a lower time constant and a closer distance to the sample can be used. This will result in fewer cycles being averaged over by the lock-in and, therefore, an increase in the overall noise. However, this also increases the maximum amplitude of the detected signal, as more of the sharpness of the peak is conserved.

In order to investigate the effect of the misalignment of the magnetic elements, for example in a microfluidic cell, a single magnetic bit is scanned with a step size of ≈3 *µ*m. The sensitive direction of the TMR sensor is aligned to the magnetic element’s long, magnetically easy, axis, and the scan bisects the element parallel to this axis. Figure 5b shows the resultant waveform. The magnitude of the field is seen to be highest close to the poles of the element. As expected, the direction of the field changes polarity over the pole itself. In the worst case scenario, this would result in a null signal in the unlikely event that the magnetic barcode flows perpendicular to the TMR sensor in a microfluidic channel. Furthermore, the bit could appear switched to the opposite state, if the microcarrier is scanned 5 *µ*m outside of the elements, as seen in Figure 5b. This imposes the restriction that the element lengths may not be miniaturised below the accuracy with which the tags may be aligned to the magnetic sensor within a microfluidic channel. However, microfluidic focusing techniques [24] can be used to ensure that the microcarriers flow centred over the detection area.

**Figure 6:**
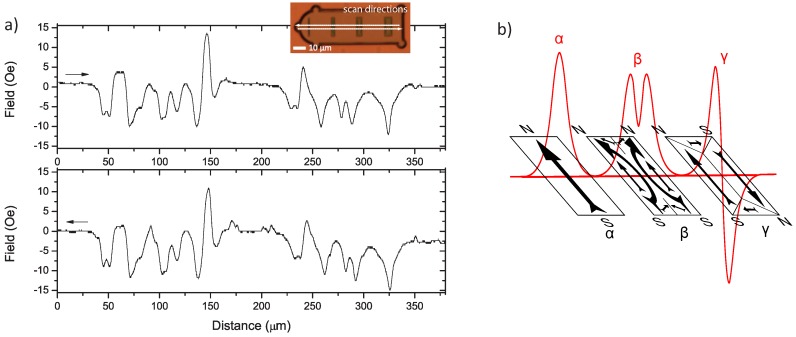
(**a**) Two four-bit tags are scanned in the forward (above) and the reverse (below) directions, showing a high level of reproducibility. The sensitive axis of the TMR sensor points along the length of the magnetic elements. A 10 *µ*m/s scan speed was used with a measurement time-constant of 100 ms. The vertical distance between the sample and sensor is ≈1 *µ*m. It is evident from the stray fields observed that the magnetic elements are not uniformly magnetised, but instead must exhibit ferromagnetic domain structures; (**b**) Schematic of three characteristic signal shapes, labelled *α*, *β* and *γ*. The corresponding distribution of edge north (N) and south (S) poles are shown along with the possible domain distributions from which these could result.

The stray fields from many of the magnetic elements indicated that the elements do not have a uniform magnetisation at remanence, but instead break down into different ferromagnetic domain configurations. The various kinds of signals observed are all present in the stray field observed from a scan of two four-bit tags shown in Figure 6a. The complexity of the signal observed renders it easy for it to be mistaken for noise; however, the return sweep reproduces the same signal, confirming that we are indeed detecting the magnetic stray field from the two tags in question. Figure 6b shows three schematics of the simplest domain configurations that would give rise to characteristic shapes, as observed from individual magnetic elements. The domain structures are speculative, with only the major pole distributions uniquely identified by our one-dimensional stray field measurement. Three characteristic signal shapes are seen:

α A single peak resulting from a single north-south (N-S) dipole. This is due to a relatively uniform magnetisation along the easy axis of the magnetic element, when the element’s width is small enough.
β A double peak resulting from two parallel N-S dipoles. This is due to the splitting of the N and S poles, as the magnetisation prefers to point towards/away from the corners rather than the flat edge. In Figure 6b, two “c”-shaped structures are assumed. Two equal abutting “c” shapes are shown, though one may dominate, resulting in a large peak and a smaller sub-peak.
γ Two peaks of opposite polarity resulting from two anti-parallel N-S, S-N dipoles. Once again, one of these dipoles may dominate, indicating the relative size of the domains, as shown in Figure 6b.

Domain breakdown generally results in a more complex signal that is harder to interpret and, in the case of the *γ* configuration, a total loss of the stored information. The domains and, therefore, the coercive field, are history and defect dependent. They effectively prevent us from reliably writing the magnetic label using a global field. This is possibly the most significant drawback of having relatively large magnetic elements.

### 4.2. Multi-Dimensional Stray Field Scans

The TMR scanning instrument is capable of much more than simple line scans. We can conduct two-dimensional scans in order to use it as an imaging tool. Magnetic sensitivity is still limited to one direction, and the resolution is limited by the size of the TMR sensor. However, using this technique, we are able to observe the structure of ferromagnetic domains by the stray field of our magnetic films.

**Figure 7:**
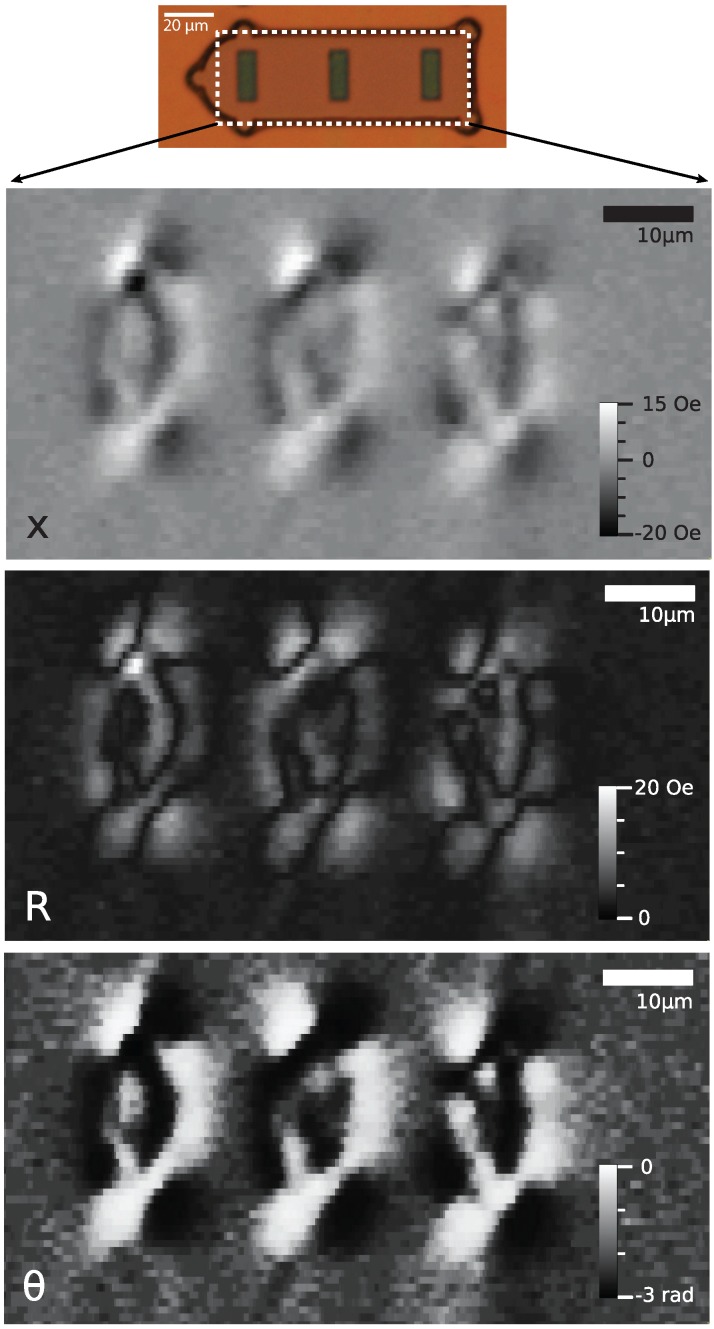
The data obtained when scanning a TMR sensor a few microns below a three-bit tag with equal aspect ratios (1:2.5) and thereby measuring the stray field in a 50 × 90 *µ*m^2^ area. The sensitive axis is vertical as presented in the figure. The images are obtained from the real component (*x*), the amplitude (*R*) and the phase (*θ*) of the signal. Frequency: 233 Hz, time-constant: 50 ms, scan speed: 40 *µ*m/s.

Figure 7 shows the images obtained when scanning a three-bit magnetic tag. The real component (*x*) of the impedance of the sensor contains information about both the amplitude and the sign of the magnetic field in the sensitive direction, which is along the length of the elements, as seen in the figure. Although one can make out that there must be a domain breakdown, it is difficult to discern the underpinning structure. This structure is much more apparent, if we display the data using the amplitude, *R*, and phase, *θ*.

The amplitude image contains no information about the sign of the signal; however, the dark lines clearly trace the domain boundaries, as they show where the stray field is zero and, therefore, changes sign. The phase image separates areas where there is a strong stray field in one direction or the other, into black and white regions, effectively revealing the sign information that is missing in the amplitude image. It can be seen that this separation of the amplitude and the sign information can be a powerful visualisation tool. We can now clearly see compelling evidence that too large magnetic elements exhibit domain breakdown.

**Figure 8:**
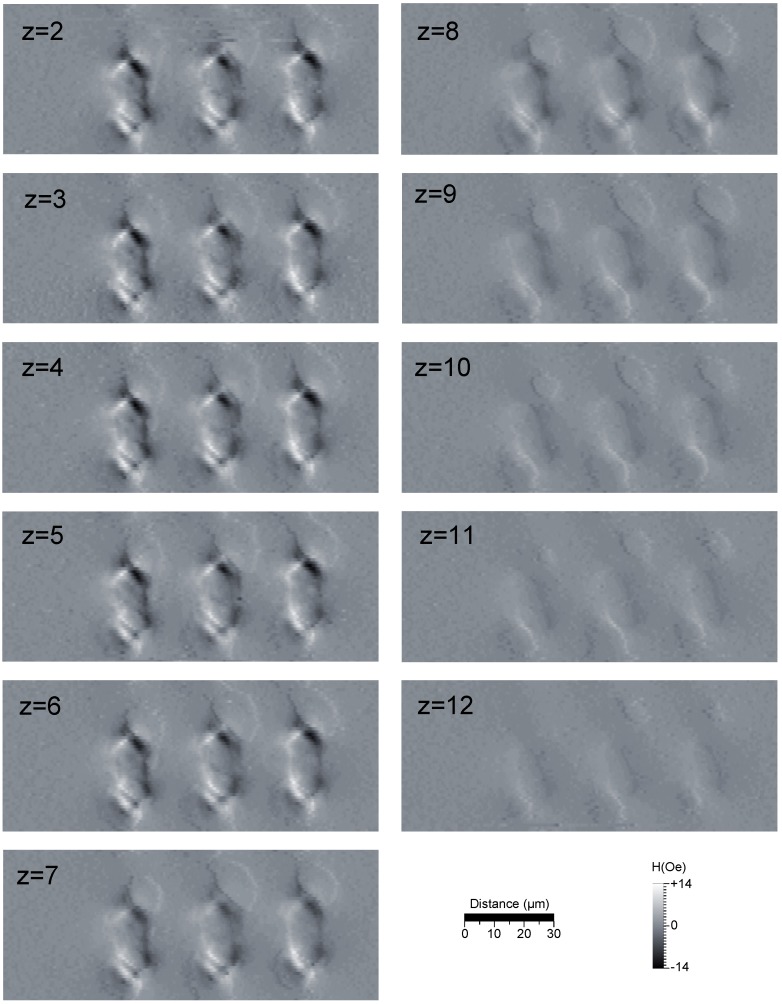
The data obtained when scanning a TMR sensor at varying heights below a three-bit tag and thereby measuring the stray field in a 50 × 150 × 10 *µ*m^3^ volume. The sensitive axis is vertical, as seen in the figure, and the images are obtained from the real component of the signal. The *z* label of each image represents the distance from the sample in *µ*m. Frequency: 833 Hz, time-constant: 50 ms, scan speed: 40 *µ*m/s.

Furthermore, our instrument can also conduct three-dimensional scans by varying the separation, *z*, between the sensor and the sample plane. Figure 8 presents the data obtained from imaging a three-bit tag from a distance of 12 to 2 *µ*m. Despite the domain breakdown, we can see that our instrument is capable of discerning a signal from the elements, even when scanning from 12 *µ*m away. However, this signal is just above the noise, and whilst it is possible to see the pattern in an image, we would not have much confidence of picking this signal out using a single-pass measurement, such as those presented in the one-dimensional scans.

## 5. Results and Discussion

The novel TMR-scanning microscope platform is capable of characterising our magnetic barcode with a *SNR* that is far superior to typical microfluidic-based detection systems for magnetically-encoded microcarriers [17]. In doing so, we determined that our tags are prone to domain breakdown, resulting in the following drawbacks:

- Loss of labelling information due to the element being in an ambiguous state.
- Attenuation of the stray field leading to difficulty in detection.
- Highly distributed stray fields due to switching dynamics.
- Broadened coercivity values of bits may result in unintentional switching of neighbouring bits during the encoding process.

These heavily limit the scaling of our technology, and as a result, we have completely reconfigured our barcode design to incorporate significantly higher aspect ratios and magnetically soft materials, in order to force domain states to be dominated by shape anisotropy. As the coercive field scales inversely with the strip width [25], the use of high aspect ratio elements significantly widens the available coercivity range for coercivity-tuned bit encoding. Moreover, by bundling groups of strips at set spacings into composite elements (CE), the dipolar interactions can be used to fine-tune a bit’s magnetisation reversal behaviour. Simultaneously, the consistent read-out properties can be maintained by keeping the magnetic volume of each bit constant. Overall, the TMR microscope has revealed key insights into our barcode design and enabled significant advances to our new CE bit design, which is presented in detail in [26].

## 6. Conclusions

We have presented a versatile stand-alone magnetic scanning instrument, outlining its design, characterisation and mapping capabilities. While it has led to significant insights into and developments for our magnetic encoding of microcarriers, the platform as a whole is also very adaptable and suitable for studying magnetic carriers of various types, such as for magnetosome imaging in magnetotactic bacteria [20] or other micron-sized magnetic entities (e.g., wires, particles, carriers). The key advantage of this set-up, as opposed to other traditional magnetometry techniques, such as magnetic force microscopy (MFM) or magneto-optic Kerr effect (MOKE) measurements, is that the TMR-scanning microscope provides quantitative magnetic field measurements enabling vector mapping of the stray field with 3D spatial resolution. We have shown that 3D raster scans can fully map out the geometrical stray fields of micron-sized magnetic elements to an accuracy of a few Oersted, limited by the detectable MR change of 0.006%/Oe. By visualising the real component, the amplitude and the phase of the sensor’s impedance, we can identify complex ferromagnetic domain structures innate to our original magnetic barcode design for microcarriers. The modular design also allows for the incorporation of different sensor heads, ranging from in-plane and out-of-plane TMR heads to giant magnetoimpedance sensors, where high-frequency band-pass filters would allow for significantly faster and higher resolution mapping of magnetic domains and stray field patterns in 3D. As a whole, the set-up can be used as a detection and characterisation tool of the variety of magnetic elements utilised in contemporary biosensor and bioassay platforms, as well as in a multitude of different scanning magnetometry applications.
